# Giant intracardiac thrombosis in an infant with leukaemia and prolonged COVID-19 viral RNA shedding: a case report

**DOI:** 10.1186/s12959-021-00285-8

**Published:** 2021-05-12

**Authors:** Ehsan Aghaei Moghadam, Shima Mahmoudi, Alieh Safari Sharari, Mehrnoush Afsharipour, Mojtaba Gorji, Amene Navaeian, Azin Ghamari, Setareh Mamishi

**Affiliations:** 1 Growth and Development Research Center, Tehran University of Medical Sciences, Tehran, Iran; 2 Department of Pediatric Cardiology, Pediatrics Center of Excellence, Children’s Medical Center, Tehran University of Medical Sciences, Tehran, Iran; 3 Pediatric Infectious Disease Research Center, Tehran University of Medical Sciences, Children’s Medical Center Hospital, Dr. Gharib Street, Keshavarz Boulevard, Tehran, Iran; 4 Pediatrics Center of Excellence, Children’s Medical Center, Tehran University of Medical Sciences, Tehran, Iran; 5 Department of Pediatrics, Pediatrics Center of Excellence, Children’s Medical Center, Tehran University of Medical Sciences, Tehran, Iran; 6 Department of Infectious Diseases, Pediatrics Center of Excellence, Children’s Medical Center, Tehran University of Medical Sciences, Tehran, Iran

**Keywords:** SARS-CoV‐2, Children, Thrombosis, Acute lymphoblastic leukemia, Prolonged viral shedding

## Abstract

**Background:**

COVID-19 can induce thrombotic disease both in the venous and arterial circulations, as a result of inflammation, platelet activation, endothelial dysfunction, and stasis. Although several studies have described the coagulation abnormalities and thrombosis in adult patients with COVID-19, there is limited data in children. Here, we present an 18-month-old boy with a prolonged SARS-CoV‐2 RNA shedding and chronic right atrial and superior vena cava (SVC) thrombosis.

**Case presentation:**

An 18-month-old boy with acute lymphoblastic leukemia (ALL) (pre-B cell ALL) and a history of chemotherapy was referred to our center due to intermittent fever with unknown origin. a positive nasopharyngeal PCR for COVID-19 was reported and stayed positive for eight consecutive weeks The high-resolution computed tomography (HRCT) showed no sign of pulmonary embolism. Initial echocardiography indicated a semilunar thrombotic mass extending from right SVC into the right atrium without coronary or myocardial involvement. Enoxaparin was administered with continuous monitoring of the level of anti-Xa activity. The serial echocardiographic studies found a slow but continuous reduction in the mass size.

**Conclusions:**

Our case shows that, as already described in adult patients, clinically relevant thrombosis can complicate the course of pediatric patients as well. In view of the specific and milder manifestations of COVID-19 in children, these complications may pose considerable diagnostic and therapeutic challenges.

## Background

The novel severe acute respiratory syndrome coronavirus 2 (SARS-CoV2), causing coronavirus disease in 2019 (COVID-19), has led to an unprecedented global health crisis.

Like adults, children can be affected by the coronavirus, but the presenting symptoms are usually milder. Prior studies have shown that angiotensin-converting enzyme II (ACE II) receptors act as a co-receptor for the virus entry into the cells [1]. This receptor has less affinity and maturity in children, which may result in children being less susceptible [2]. However, several studies have demonstrated that children can be affected by COVID19 [3, 4].

Although lung is the primary site of SARS-CoV2, the virus can disseminate to different organs and induce even atypical pathological conditions [5]. COVID-19 can induce both venous and arterial thrombosis, as well as myocardial injury, arrhythmia, and acute coronary syndrome, as a result of inflammation, platelet activation, endothelial dysfunction, and stasis [6–9].

Although several studies have described the coagulation abnormalities and thrombosis in adult patients with COVID-19, there is limited data in children. Here, we present an 18-month-old boy treated for acute lymphoblastic leukemia (ALL) with prolonged SARS- CoV-2 RNA shedding and chronic right atrial and superior vena cava (SVC) thrombosis.

## Case presentation

An 18-month-old boy with pre-B cell ALL currently 3 weeks after the maintenance chemotherapy with vincristine PEG-asparaginase, methotrexate, doxorubicin and dexamethasone was referred to our center due to intermittent fever with unknown origin. Other symptoms included rash, diarrhea, vomiting, cough, or coryza. Physical examination revealed paleness without any specific symptom for infection. There was no other finding in favor of systemic or pulmonary embolism.

Laboratory tests revealed low level of white blood cell count, hemoglobin, and platelet. Our patient had elevated markers of thrombosis, inflammation, and cardiac injury. High levels of D-dimer (4000 pg/ml; day 6), fibrinogen (647 mg/dl; day 12), and fibrin degradation product (FDP) (10 ng/ml; day 12) were observed. Lactate dehydrogenase (LDH) level was 1518, 1272, 866 and 622 U/L at day 1, 3, 6, and 12, respectively. Moreover, elevated level of erythrocyte sedimentation rate (ESR) and C-reactive protein (CRP) were observed (Table 1).

**Table 1:** Laboratory findings of the patient in different times

	Day 1	Day 3	Day 6	Day 12	Day 40	Day 60
White blood cell count (× 10^9^ cells per L)	0.3	0.8	3.6	4.6	2.1	2.9
Neutrophil count (× 10^9^ cells per L)	-	-	2.38	3.78	0.75	1.72
Lymphocyte count (× 10^9^ cells per L)	-	-	0.18	0.19	0.61	0.44
Haemoglobin (g/dL)	9.1	8.7	9.2	11.3	9.1	10.6
Platelet count (× 10^9^ cells per L)	17	50	254	51	94	90
D-Dimer (pg/mL)	-	-	4000	-	200	-
Fibrinogen (mg/dL)	-	-	-	647	307	-
Interleukin 6 (pg/mL)	-	-	257	-	98	-
Fibrin degradation product (FDP) (ng/ml)	-	-	-	10	4	-
Erythrocyte sedimentation rate (mm/h)	69	-	-	42	24	-
C-reactive protein (mg/L)	52	-	-	75	26	-
Aspartate aminotransferase (U/L)	53	-	-	13	24	19
Alanine aminotransferase (U/L)	40	-	-	6	5	5
Lactate dehydrogenase (U/L)	1518	1272	866	622	-	-
Creatinine (µmol/L)	0.3	0.4	0.4	0.3	0.3	0.4
Prothrombin time (s)	-	-	13.3	-	13.4	-
Partial thromboplastin time (s)	-	-	30	-	43	-
International normalized ratio	-	-	1.1	-	1.11	-
Blood culture	Positive	-	-	Negative	Negative	Negative
SARS-CoV-2 rRT-PCR	-	Positive	-	Positive	Positive	Positive

The blood culture was positive for *Pseudomonas* Spp. Due to the patient’s critical condition, antibiotic therapy was initiated with meropenem and vancomycin and continued for four weeks.

Nasopharyngeal samples were collected and tested for SARS-CoV-2 using the Reverse transcription polymerase chain reaction (rRT-PCR) assay according to the previous report [1]. Meanwhile, a positive nasopharyngeal PCR for COVID-19 was reported and stayed positive for eight consecutive weeks (Table 1). The high-resolution computed tomography (HRCT) showed no sign of pneumonia or pulmonary embolism. A portal venous catheter was implanted for anti-cancer therapy, and adequate anticoagulant therapy was administered after the diagnosis of the thrombosis was diagnosed.

**Fig. 1:**
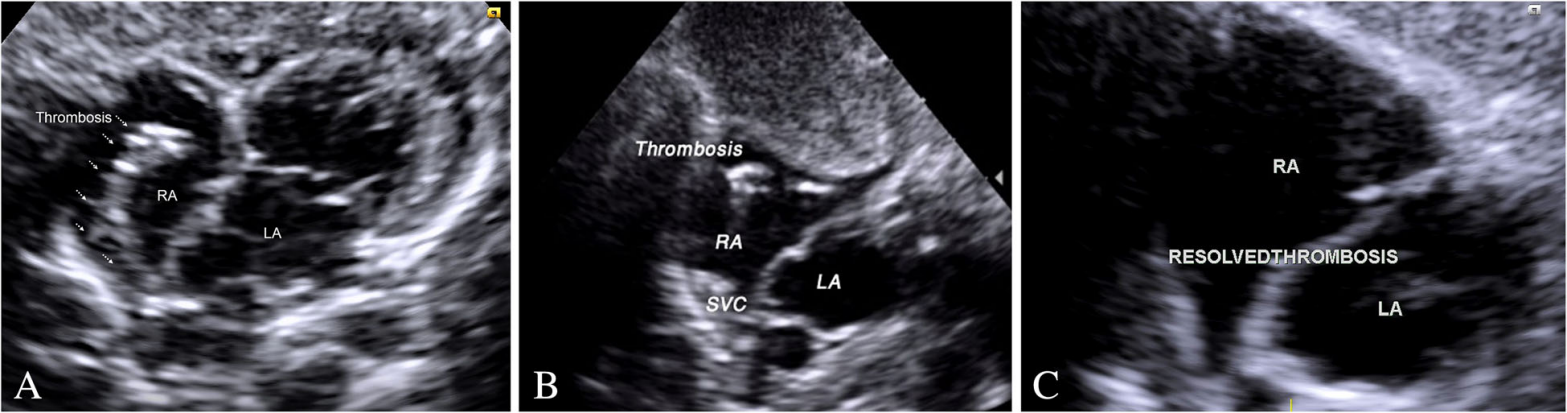
Serial echocardiography **a** First echocardiography, crescentic thrombotic mass in the right atrium, **b** After 2 months of enoxaparin treatment, **c** Four-month follow-up. IVC: inferior vena cava, SVS: superior vena cava, RA: right atrium, LA: left atrium, LV: left ventricle, RV: right ventricle

Initial echocardiography indicated a semilunar thrombotic mass extending from right SVC into the right atrium without coronary or myocardial involvement (Fig. 1). The patient was a candidate for surgical mass removal due to increased risk for thromboembolic events; however, the intervention was postponed because of his unstable condition.

Enoxaparin was administered with continuous monitoring of the level of anti-Xa activity. The serial echocardiographic studies found a slow but continuous reduction in the mass size (Fig. 1); therefore, surgical removal of the thrombosis was cancelled in favour of anticoagulation therapy with medical follow-up. After 4 months, follow-up echocardiography was performed and the result indicated that the thrombosis was completely resolved (Fig. 1).

Follow-up SARS-CoV-2 testing was performed on day 3, 12, 40 and 60 with the goals of removing isolation precautions, and supporting appropriate discharge planning and all test were positive for SARS-CoV-2 nasopharyngeal rRT-PCR of SARS-CoV-2.

## Discussion and conclusions

To the best of our knowledge, this is the first report describing giant intracardiac thrombosis following the COVID 19 infection in a child with ALL and prolonged SARS-CoV‐2 RNA shedding.

Typically, SARS-CoV-2 RNA levels are detectable in the respiratory tract 2–3 days before symptoms onset, decline over the following 7–8 days and become nondetectable thereafter [10]. The time that viral RNA remains positive is not well understood. However, some studies have reported viral shedding for up to 3 months after resolve of symptoms [11–13].

Cancer patents are immunologically naive to this novel virus and are at risk of a more severe course of COVID-19. They generally shed community-acquired respiratory viruses longer than immunocompetent people due to a constrained immune response [14, 15]. In addition, the risk of thrombosis associated with ALL may add to that imposed by COVID-19. Lehners et al. reported the long-term virus detection for more than 30 days in 29 % of infected patients with respiratory virus infections [14].

Our case had experienced a prolonged duration of viral shedding; however, it is unclear whether this represents infectious virus and poses a risk for forward transmission or not. Finally, we were unable to determine the exact duration of RT-PCR positivity in this case,

COVID-19 causes coagulation abnormalities in a proportion of patients, which can lead to thromboembolic events [7, 16]. According to the latest Meta-analysis report [17], adult patients with severe COVID-19 infection had higher levels of D-dimer and fibrinogen and thrombo-inflammatory biomarkers including D-dimer, CRP, and LDH may predict the poor prognosis and severity of COVID-19 infection. Although a majority of patients with COVID-19 with coagulation abnormalities did not have the criteria of disseminated intravascular coagulation, diverse cardiovascular manifestations of COVID-19 observed [8]. Since our patient was not at risk for hypercoagulability due to the low platelet levels and bone marrow dysfunction, prolonged COVID-19 infection might be related to the persistent thrombosis.

The mechanisms of these coagulation abnormalities, following COVID-19 are unclear. However, severe inflammatory response as well as endothelial damage following COVID-19, particularly in cases with underlying comorbidities might predispose patients to a hypercoagulable conditions [18, 19].

Diagnosing of thrombosis may be more difficult in children with COVID-19 because of their milder clinical presentation. However, more research is needed in the prognostic value of monitoring the dynamics of coagulation parameters and ther prognostic value in pediatric patients as well, as shown in adult COVID-19 populations [20]. According to the consensus-based clinical recommendations, the use of anticoagulant thromboprophylaxis in hospitalized children with COVID‐19‐related infection is suggested [21]; however, more guidance is needed in determining the need and modalities of both prophylactic and therapeutic anticoagulation in pediatric patients [22].

Caution is needed when drawing implications from our case. Despite the prolonged duration of viral shedding in our case, we were unable to determine the exact duration of RT-PCR, nor whether this was associated with infectivity and risk for transmission. However, we should mention the limitation on the relative contribution of the three risk factors including ALL, COVID-19 and, to a smaller extent, Pseudomonas infection.

In conclusion, our case shows that, as already described in adult patients, clinically relevant thrombosis can complicate the course of pediatric patients as well. In view of the specific and milder manifestations of COVID-19 in children, these complications may pose considerable diagnostic and therapeutic challenges. 

## Data Availability

All data obtained.

## Abbreviations

SARS-CoV2: Severe acute respiratory syndrome coronavirus 2
COVID-19: Coronavirus disease 2019
SVC: Superior vena cava
ALL: Acute lymphoblastic leukemia
rRT-PCR: Reverse transcription polymerase chain reaction
HRCT: High-resolution computed tomography
